# Progress of Medical Science in Germany

**Published:** 1874-12-01

**Authors:** Edmund J. Doering


### PROGRESS OF MEDICAL SCIENCE IN GERMANY.

By Edmund J. Doering, M.D.

I. Treatment of Diphtheria and Scarlet Fever, by Dr. G. Meyer (*Jahrb. f. Kinderheilk.*, vii, 4). II. A Case of Displacement of the Spleen, by Dr. Pirotais (*Arch. Med. Belges and Med. Centr. Zeitüng*, No. 86). III. Intermittent Nightly Hæmoptysis of Consumptives, by Dr. C. Gerhardt (*Deutsche Z. f. pr. Med.*, No. 43, 1874).

### I.

Dr. Meyer is strongly in favor of the treatment of diphtheria with ice. Even in children under the age of one year he directs small pieces of ice to be put frequently into the mouth, followed, if possible, every minute or two, by a teaspoonful of ice water. The ice must be pure, and therefore ice artificially prepared is preferable. In severe cases the external use of cold, by means of an ice bag applied around the throat, is very useful. The author has found that by this mode of treatment the fever soon diminishes and the diphtheritic membrane is detached and expectorated. It is only in exceptional cases that the disease extends nevertheless to the larynx. In but one case the author was obliged, in order to reduce the temperature, to resort to cool baths. The latter he also found of great service in scarlet fever. Whenever the temperature exceeds 102° in scarlet fever the patient is to be placed for ten minutes in a bath of a temperature varying from 93° to 73°, according to the intensity of the fever. The effect of these baths in reducing the temperature lasts for several hours.

### II.

Various causes may produce displacement of the spleen. These are either physiological, as the respiratory act, or morbid, as hydrothorax, ascites, abdominal tumors, and abnormal conditions of the splenic ligaments.

Different observers have reported interesting cases of this sort, viz.: Choisy, who found the spleen freely movable in the abdominal cavity; Matter likewise in the regio-iliaca and hypogastrica; Faudacy, in the inguinal region; Van Swieten in the pelvis, and, finally, Devergie, who found the spleen in the thorax after rupture of the diaphragm.

A sudden displacement of the spleen is of rare occurrence, on account of the strength of the ligamenta phrenosplenica and gastrosplenica, which are not easily torn, as can be demonstrated on the cadaver. But that it may occur, is shown by the following case:

A lady, thirty-five years old, who had always been healthy, was injured by a fall from a wagon, which produced great pain in the left hypochondriac region, vomiting, and inability to move. The attending physician found a tumor at the site of pain, to which he applied leeches and friction, with mercurial ointment, but the pain and vomiting continued.

The writer was called in to see her about six weeks after the accident occurred. On examination he found a somewhat movable, circumscribed tumor, in the left iliac region, six inches in length and three and a half inches in breadth. Pulse 80; temperature normal. He diagnosed the tumor to be the spleen and returned it to the left hypochondriac region, retaining it there by a compress and a firm binder.

After the reduction the pain and vomiting subsided. A few days later the patient was able to retain food and to leave her bed, to which she had been confined ever since the accident.

III.

Observation of the bodily temperature in phthisis pulmonalis discloses a number of interesting facts. It will be found that quite a number of patients are for a long time free from fever, while others again, from the slightest complication, have a high temperature. If the measurements are made frequently, about every three hours, one observes that the fever is of an intermittent type in the majority of patients. Toward the last stage of the disease in patients who are much exhausted, the morning temperature is often below normal, at times as low as 97° F.; not rarely the fever takes on a typus inversus for one or more days, *i. e.*, morning exacerbations and evening remissions. Jochman believes this to be due to complications. The author has observed this type of fever most generally in patients who did not rest well the night previous. He remembers especially one instance, when, after a severe thunderstorm during the night, more than half of the numerous consumptives in his wards showed an increase of temperature in the morning. The remission of the fever occurs generally during the hours from midnight till morning, the temperature rising in the forenoon and reaching its maximum height towards evening.

The daily chill of many consumptives corresponds to the rise, and the night sweats to the fall of the temperature. During the fever the vascular pressure diminishes, the arteries relax and widen, the secretions are diminished, and there is a retention of water in the system. Vice versa during the remission, the vascular pressure increases, the arteries contract, and the surplus quantity of water is eliminated in the form of profuse sweating. Now, in many consumptives, there is a morbid friability of the smaller blood-vessels, which are just able to resist the diminished vascular pressure during the fever, but not the increased pressure during the remission, hence rupture and hæmorrhage result. To this fact the author attributes the nightly attacks of hæmoptysis occurring in two of his patients for several nights in succession.

The patients were sleeping quietly and perspiring freely when they were suddenly awakened by a desire to cough, accompanied by the expectoration of bright red blood, varying in quantity from a tablespoonful to a small spittoonful. The average quantity each time was from three to six ounces. These hæmorrhages were distinguished by their regular return during the remission of the fever at night, and the author therefore justly terms them attacks of nightly intermittent hæmoptysis. In the above cases the usual hæmostatics were given, as tannin in large doses, ergot, mineral acids and inhalations of the chloride of iron, but without success. The author then gave large doses of quinine with the view of breaking up the fever, in order to diminish the fluctuations in the vascular pressure. The result established the correctness of the author's views, for the hæmorrhages were thus stopped at once. A subsequent return of the hæmorrhage subsided likewise on the renewed administration of fifteen grains of quinine, given in the morning. One of the patients finally succumbed to the extension of the disease. The other regained sufficient strength to travel home.

The author also believes that hæmorrhages occurring during the remission of other fevers ought to be considered from this standpoint.

He has seen several cases of sudden death of very old men during the crisis of pneumonia, from cerebral hæmorrhage. The author therefore concludes that special attention ought to be paid to the proper elimination of the secretions during the crisis of pneumonia, and, when retarded, we should take into consideration the propriety of performing venesection.

